# Supplementary material for: Changes in the secondary compounds of persimmon leaves as a defense against circular leaf spot caused by Plurivorosphaerella nawae
Source: PLoS One. 2020 Mar 6;15(3):e0230286. doi: 10.1371/journal.pone.0230286 (PMC7059969; doi:10.1371/journal.pone.0230286)
Supplement: Attachment — Submitted filename: Response to Reviewers.docx [file pone.0230286.s002.docx]

Dear reviewers

Thank you very much for your query and suggestions.

We tried to address all the concerns of you.

The response of all point raised by reviewer 1 is given bellow-

- In the article, there is described a holistic response of persimmon leaves on the infection of P. nawae. The experiment is well designed but I have some doubts about analyzes of metabolites. In line 111 cited is a method that is not listed in the references. The drying and blanching at 100 °C could influence the content of phenolics and especially ascorbic acid. Furthermore, vitamin C includes L-ascorbic acid and dehydroascorbic acid, not only ascorbic acid. And a temperature range of 30 to 60 degrees C could result in the conversion of L-ascorbic acid to dehydroascorbic (DHAA), a very important reaction in regard to vitamin C degradation because DHAA could be easily converted to other compounds that do not have the biological activity of vitamin C (Munyaka et al., 2010, J Food Sci.; Herbig and Renard, 2017, Food Chem.).
  When the units are mg/g we do not use the term “concentration” but “content”. The term “concentration” is used when the amount is present in volume.
- The leaves used for Vitamin C were not balanced. They were freeze-drying for 72 h in a vacuum freeze dryer. We rewrite concentration as content.
- Vitamin C - unifrom the writing
- Done
- Line 105: define more specific when infected leaves were collected
- Defined
- Line 129: referencing is not appropriate
- Corrected
- Line 130: a comma is missing
- Corrected
- Line 157: why for Acontrol the DPPH solution was used and not blank (sample replaced by water+MeOH)?
- we used DPPH solution as a control because we wanted to see its effect as DPPH change the color when neutralized. This a standard method. see reference (20,34)
- Line 175: … filtered using Bucher. Explain more precisely what it is Bucher.
- Explained
- Line 206: what is meaning “r. AUDPC” ?
- R package ‘stats’ (version 3. 4.1). AUDPC= Area under the disease progress curve. It was fixed in text wit reference.
- Line 318: What did you mean with “phenols” in the statement: “… phenolic compounds (phenols, phenolic acids, flavonoids, …)?
- Corrected as total phenolics
- Line 332: referencing is not appropriate
- Corrected
- References have to be checked detailed. Many of them are not correct used in the text. In the part References some are missing (Hossain et al., 2017), some are written twice (Mikulic-Petkovsek et al., 2011).
- Checked

I hope everythings were made clear and understandable.

Thank you for your time.

Your best regard

Chang
