# Supplementary material for: Changes in the secondary compounds of persimmon leaves as a defense against circular leaf spot caused by Plurivorosphaerella nawae
Source: PLoS One. 2020 Mar 6;15(3):e0230286. doi: 10.1371/journal.pone.0230286 (PMC7059969; doi:10.1371/journal.pone.0230286)
Supplement: Attachment — Submitted filename: Response to Reviewers.docx [file pone.0230286.s003.docx]

Dear Editor

Thank you very much for your query and suggestions.

We tried to address all the concerns of you.

The response of all point raised by reviewer 1 is given bellow-

- In the previous review round, authors were addressed to meticulously check and revise the manuscript according to reviewers' comments. Reviewer #1 have raised major concerns about the methodology used, particularly related to the plant material handling and temperature-dependent degradation of L-ascorbic acid. More thorough discussion both in the main text and in "response to reviewers" were expected. The claims stated in the response to the reviewer's concerns have to be supported by a steady literature survey, whether the authors agree with the comments or provide a rebuttal.
- Actually, there was a misunderstanding. Reviewer thought the leaves used for vitamin C analysis were dried and blanched at 100 °C. Obviously L-ascorbic acid degraded at hi temperature. But the leaves used for Vitamin C in this study were not balanced. They were freeze-drying for 72 h in a vacuum freeze dryer at -50 °C. we followed the exact methods that followed by one of our author Abul Hossain. Please see reference (20,34)
- There is no need to capitalize the term "vitamin".
- Corrected
- Wrong figure legend provided for Figure 5 in the Figure legends list.
- No. Figure 5 and 6 looks like same but they result of two experiment. Figure 5 is for field experiment and Figure 6 for control experiment
- L143: no need to state the full compound name in the subtitle.
- Corrected
- A brief conclusion part should be provided.
- A brief conclusion part has been added

I hope everything were made clear and understandable.

Thank you for your time.

Your best regard

Chang
